# MR-Neurography of the sural nerve in patients with hereditary amyloidosis

**DOI:** 10.1186/1750-1172-10-S1-P40

**Published:** 2015-11-02

**Authors:** Jennifer Kollmer, Markus Weiler, Jan Purrucker, Ute Hegenbart, Stefan Schönland, Christoph Kimmich, Martin Bendszus, Mirko Pham

**Affiliations:** 1 University of Heidelberg, Department of Neuroradiology, 69120, Heidelberg, Germany; 2 University of Heidelberg, Department of Neurology, 69120, Heidelberg, Germany; 3 University of Heidelberg, Medical Department V (Amyloidosis Center), 69120, Heidelberg, Germany

## Background

Sural nerve biopsies are often performed in order to detect the underlying disease in patients suffering from unclear polyneuropathic symptoms. In transthyretin familial amyloid-polyneuropathy (TTR-FAP) the diagnostic value of invasive sural nerve biopsies is controversially discussed as it often lacks to detect amyloid deposits [Simmons et al, J Neurol Sci 1993]. As we recently reported, amyloid related nerve-injury in TTR-FAP can be unambiguously determined in large-caliber nerves (sciatic, tibial and common peroneal nerve) by applying high-resolution MR-Neurography (MRN) [Kollmer et al, Brain 2015]. However, the diagnostic yield of MRN of the small-caliber sural nerve, representing the target nerve specimen for biopsies, is still unclear and was subject to this investigation.

## Methods

We prospectively enrolled 25 patients with manifest TTR-FAP, 10 asymptomatic gene-carriers with confirmed mutations in the TTR-gene, and 40 age/gender-matched healthy volunteers. Besides detailed neurological and electrophysiological examinations in all patients, a sural nerve biopsy was obtained in 12/25 manifest TTR-FAP patients. All participants underwent the following high-resolution MRN protocol (3Tesla/Magnetom/TIM-TRIO/Siemens):1) axial 2D-T2-TSE-fs (TR/TE 5970/55ms, voxel-size 0.4x0.3x3.5mm^3^); 2) axial 2D-dual-echo-TSE-fs (TR 5210ms, TE1/TE2 12/73ms, voxel-size 0.4x0.3x3.5 mm^3^).

On each axial imaging slice the sural nerve was identified and manually segmented. After signal-normalization (histogram-based, comparison with control population), nerve-voxels were statistically classified as nerve-lesion-voxels by operator-independent, threshold-based segmentation. The apparent-T2-relaxation-time and proton-spin-density were calculated for all nerve-lesion-voxels.

## Results

Sural nerve lesion-voxels were found to be significantly higher in manifest TTR-FAP vs. controls (p<0.0001), in asymptomatic gene-carriers vs. controls (p<0.0001) and in manifest TTR-FAP vs. asymptomatic carriers (p=0.0035). Wilcoxon rank-sum-test revealed with high statistical significance that proton-spin-density was higher in severely affected TTR-FAP patients (p<0.0001), in moderate TTR-FAP (p<0.0001) and also in asymptomatic gene-carriers (p=0.0003) compared to healthy controls. The apparent-T2-relaxation-time was significantly increased in symptomatic TTR-FAP (p<0.05) but not in asymptomatic gene-carriers (p=0.4286) compared to controls.

## Conclusion

MRN of the sural nerve is a new, non-invasive and highly sensitive diagnostic tool, which can clearly differentiate between symptomatic TTR-FAP, asymptomatic gene-carrier status and healthy controls by evaluating nerve-lesion-voxels and proton-spin-density. Additional analyzes of the apparent-T2-relaxation-time can further confirm symptomatic disease. Results of this evaluation may have a strong impact for a better diagnostic interpretation of negative sural nerve biopsies.

